# Inflammatory and endothelial dysfunction indices among Egyptian females with obesity classes I–III

**DOI:** 10.1042/BSR20192910

**Published:** 2020-09-21

**Authors:** Amal Ahmed Mohamed, Wafaa Gh. Shousha, Moushira Erfan Zaki, Hala T. El-Bassyouni, Hadeel El-Hanafi, Sara M. Abdo

**Affiliations:** 1 Biochemistry Department, National Hepatology and Tropical Medicine Research Institute, Cairo, Egypt; 2 Chemistry Department, Division of Biochemistry, Faculty of Science, Helwan University, Cairo, Egypt; 3 Biological Anthropology Department, National Research Centre, Cairo, Egypt; 4 Clinical Genetics Department, National Research Centre, Cairo, Egypt; 5 Clinical and Chemical Pathology Department, Faculty of Medicine, Cairo University, Egypt

**Keywords:** body mass index, endothelial dysfunction, IL-12, inflammation, Obesity, TNF-α

## Abstract

**Background:** Obesity is an alarming threat to health in Egypt. More than one in three Egyptians is obese, the highest rate in the world. We aimed to delineate the variability of inflammation and endothelial dysfunction markers among Egyptian females with different obesity classes. **Methods:** Out of 130 females, 70 were categorized into three obesity groups: Class I, body mass index (BMI) 30–34.9 kg/m^2^; Class II, BMI 35–39.9 kg/m^2^ and Class III BMI ≥ 40 kg/m^2^, besides 60 control subjects. Anthropometric measurements were recorded and serum levels of tumor necrosis factor-α (TNF-α), C-reactive protein (CRP), interleukin (IL) 6 (IL-6), IL-12, soluble intercellular adhesion molecule 1 (sICAM-1) and soluble vascular adhesion molecule 1 (sVCAM-1) were assessed among participants. **Results:** In all three classes of obesity, significant increase (*P* <0.05) in BMI, waist-hip ratio, fat mass and body fat mass % were noted. CRP and sVCAM-1 levels were increased among the three obesity groups. TNF-α levels were increased in class II and III obesity groups. IL-6 and IL-12 levels were elevated in class I and class III groups. While, ICAM-1 levels were increased in class III obesity group. **Conclusion:** Based on individuals’ BMI, serum levels of TNF-α, CRP, IL-6, IL-12, sVCAM-1 and sICAM-1 are differentially altered with the progression of obesity. We strongly support the hypothesis that, as the obesity rate is still mounting, a subclinical inflammatory reaction has a role in pathogenesis of obesity and emphasize the elevation of endothelial dysfunction in individuals with obesity.

## Introduction

Obesity is an alarming threat to health in Egypt causing harm to both individuals and society. The estimated prevalence of overweight and obesity (body mass index (BMI) ≥ 25 kg/m^2^) is 61–70% of the whole population aged 20 and above [1]. As for obesity alone, analyzed data from 68.5 million people across 195 countries and territories indicated that, 35% of Egyptian adults (∼19 million Egyptians) suffer from obesity—the highest rate in the world [2].

An individual develops obesity in case of persistent imbalance between energy intake and expenditure, as a consequence of complex interaction of genetic, dietary, environmental and social factors [3]. No matter the stage of development, obesity affects both the quality of people’s lives and may also shorten their lives [4]. Indeed, obesity facilitates the development of metabolic disorders (e.g. diabetes, hypertension), and cardiovascular diseases (CVDs) in addition to chronic diseases (e.g. stroke, osteoarthritis, sleep apnea, cancers and inflammation-based pathologies) [5,6].

Inflammation has been implicated in the pathophysiology of individuals with obesity. The initial factors involved in generating the inflammatory events in the obesity remain unclear and controversial [7]. It is plausible that is related to expansion of adipocytes and infiltration of macrophages into adipose tissue (AT), where there is an increased secretion of pro-inflammatory cytokines such as interleukin (IL)-6 (IL-6), IL-8, tumor necrosis factor (TNF-α), complement C3 and monocyte chemoattractant protein-1 (MCP-1) [7–9]. These pro-inflammatory cytokines can also affect insulin sensitivity and endothelial dysfunction, as well as stimulate a proliferative response in the vascular wall, which clearly promotes an increased risk for numerous comorbidities such as type 2 diabetes (T2D) and CVDs [10].

In the present study, we aimed to investigate the variability of inflammatory and endothelial cell dysfunction markers among Egyptian females with obesity classes I–III.

## Methods

### Subjects

The study enrolled 130 females, of which 70 were classified into three obesity groups: Class I obesity with BMI of 30–34.9 kg/m^2^ (*n*=24); Class II obesity with BMI of 35–39.9 kg/m^2^ (*n*=23) and Class III obesity with BMI ≥ 40 kg/m^2^ (*n*=23), according to WHO published data [11], in addition to 60 normal subjects as a control group (BMI < 25 kg/m^2^). Subjects were recruited from the National Research Centre (NRC), Cairo, Egypt. The study protocol was approved by the Medial Ethics Committee of the NRC and the informed consents for experimentation were obtained from the participants. The selection criteria included: they were older than 18 years, did not have a diagnosed CVD, liver, kidney, central nervous system, endocrine and/or metabolic disorders, as well as that they were not under a medical drug therapy that could affect the level of lipids and glycemic regulation.

### Anthropometric measurements

The anthropometric measurements were recorded for all the subjects: body weight (kg), body height (cm), BMI were determined based on these results, waist and hip circumference (cm), as well as the ratio between waist and hip circumference (WC/HC) and body fat mass were assessed by Tanita Body Composition Analyzer.

### Biochemical analyses

Blood samples were collected and the sera were separated by cooling centrifugation (4°C) at 1800×***g*** for 10 min and then stored immediately at −80°C till analysis. The serum concentration levels of TNF-α, C-reactive protein (CRP), soluble vascular adhesion molecule 1 (sVCAM-1), soluble intercellular adhesion molecule 1 (sICAM-1), IL-6 and IL-12 were assessed by enzyme-linked immunosorbent assay (ELISA) according to the manufacturer’s instructions. The concentration of TNF-α was assayed using human TNF-α ELISA [12]; CRP was estimated with human CRP ELISA [13]; sVCAM-1 and sICAM-1 were assayed with humans VCAM-1 and ICAM-1 ELISA (IBL International GmbH, Hamburg, Germany); IL-6 and IL-12 were determined using human IL-6, IL-12 ELISA (Quantikine R&D Systems, Minneapolis, U.S.A.).

### Statistical analysis

Data are presented as mean ± standard deviation (SD). All analyses were conducted via IBM SPSS Statistics program, Version 23. Data were analyzed using one-way analysis of variance (ANOVA), followed by least significant difference (LSD) to compare significance between groups. Statistically significant values are considered at *P*-value <0.05.

## Results

Table 1 shows the anthropometric parameters of the studied participants compared with those of the control group. Significantly increased levels of BMI, body weight, waist-hip ratio (WHR), fat mass and body fat mass % were noted in the three obesity groups as compared with controls.

**Table 1:** Anthropometric characteristics of the studied participants

Characteristics	Normal control (*n*=60)	Class I obesity (*n*=24)	Class II obesity (*n*=23)	Class III obesity (*n*=23)	*P*-value
**Age (years)**	24.8 ± 4.3	25.82 ± 5.5	26.21 ± 6.9	26.78 ± 5.3	P1 = 0.02
					P2 = 0.01
					P3 = 0.001
**Weight (kg)**	53.1 ± 15.2	94.4 ± 11.4	99.3 ± 8.7	113.2 ± 12.6	P1 = 0.0001
					P2 = 0.0001
					P3 = 0.0001
**BMI (kg/m^2^)**	21.2 ± 4.72	33.9 ± 0.74	37.2 ± 1.06	47.7 ± 19.4	P1 = 0.0001
					P2 = 0.0001
					P3 = 0.0001
**WC (cm)**	72 ± 8.43	106.1± 5.22	107.4 ± 7.6	116 ± 9.6	P1 = 0.0001
					P2 = 0.001
					P3 = 0.0001
**HC (cm)**	94.1 ± 13.4	120.8± 5.09	124.34 ± 5.7	136.2 ± 9.5	P1 = 0.001
					P2 = 0.001
					P3 = 0.0001
**Fat mass**	13.9 ± 3.01	37.6± 2.5	43.5 ± 4.01	53.7 ± 9.6	P1 = 0.001
					P2 = 0.0001
					P3 = 0.0001
**Fat mass %**	21.33 ± 2.11	29.67 ± 2.29	42.5 ± 3.78	48.67 ± 4.98	P1 = 0.003
					P2 = 0.001
					P3 = 0.0001
**WC/HC**	0.79 ± 0.06	0.83 ± 0.04	0.89 ± 0.06	0.91 ± 0.08	P1 = 0.003
					P2 = 0.001
					P3 = 0.0001

Data are expressed as mean ± SD. Abbreviations: HC, hip circumference; WC, waist circumference. P1, P2, P3 represent statistically significant *P*-values in comparison among classes I, II and III obesity groups and control group, respectively.

Regarding the inflammatory markers, we observed significantly elevated levels of CRP among the three obesity groups versus the normal control group. On the other hand, TNF-α levels were significantly increased in class II and III obesity groups only, while statistically no significant change has been noted at TNF-α levels in class I obesity in comparison with the normal subjects. Also, significant increase in levels of IL-6 and IL-12 among class I and class III obesity groups with respect to normal control were detected. In contrast, class II obesity showed no statistically significant change in levels of IL-6 and IL-12 versus normal control group (Table 2).

**Table 2:** Inflammatory biomarkers among obesity classes versus normal lean

	Normal control (*n*=60)	Class I obesity (*n*=24)	Class II obesity (*n*=23)	Class III obesity (*n*=23)	*P*-value
**TNF-α (ng/ml)**	92.6 ± 2.2	95.8 ± 2.1	106.2*^,†^ ± 2.5	124.8*^,†^ ± 4.8	P1 = 0.46
					P2 = 0.003
					P3 = 0.000
**CRP (mg/dl)**	4.5^†^ ± 0.4	7.5* ± 1.06	9.17* ± 0.9	8.5* ± 0.9	P1 = 0.02
					P2 = 0.001
					P3 = 0.002
**IL-6 (pg/ml)**	93.8^†^ ± 4.1	140.5* ± 14.6	116.1 ± 7.3	130.5* ± 8.0	P1 = 0.001
					P2 = 0.09
					P3 = 0.007
**IL-12 (pg/ml)**	140.6^†^ ± 8.1	170.9* ± 10.8	165.3 ± 10.7	181.8* ± 11.8	P1 = 0.04
					P2 = 0.1
					P3 = 0.006

Data are expressed as mean ± SD. (*): Statistically significant values (at *P*<0.05). P1, class I obesity vs control; P2, class II obesity vs control; P3, class III obesity vs control. (^†^): Values statistically significant (*P*<0.05) in comparison with class I obesity group (BMI 30–34.9 kg/m^2^).

Serum levels of sVCAM-1 exhibited significant increase among the three obesity groups in comparison with control. On the other side, only class III obesity group recorded significant increase in sICAM-1 levels as compared with normal control (*P*=0.0001), while ICAM-1 levels did not differ statistically in the initial stages of obesity (class I and II obesity groups) (Table 3).

**Table 3:** Endothelial cell markers among obesity classes versus normal control

	Normal control (*n*=60)	Class I obesity (*n*=24)	Class II obesity (*n*=23)	Class III obesity (*n*=23)	*P*-value
**sVCAM-1** (ng/ml)	95.6^†^ ± 1.8	110.6* ± 5.7	111.1* ± 4.3	124.4*,^†^ ± 4.3	P1 = 0.01
					P2 = 0.01
					P3 = 0.000
**sICAM-1** (ng/ml)	96.3 ± 2.4	106.5 ± 5.2	111.2 ± 6.7	156.1*^,†^ ± 12	P1 = 0.33
					P2 = 0.16
					P3 = 0.0001

Data are expressed as mean ± SD. (*): Statistically significant values (at *P*<0.05); P1, class I obesity vs control; P2, class II obesity vs control; P3, class III obesity vs control. (†): Values statistically significant (*P*<0.05) in comparison with class I obesity group (BMI 30–34.9 kg/m^2^).

## Discussion

Obesity is known as a perpetual state of chronic low-grade inflammation, through systemic and paracrine increase in levels of cytokines and chemokines [14,15]. In the current study, the serum levels of TNF-α, CRP, IL-6, IL-12, sVCAM-1 and sICAM-1 were evaluated with the progression of obesity classes I–III. Several reports have mainly described the inflammatory events in individuals with obesity, but limited, controversial and inconclusive results were observed with particular reference to obesity classes. Up to date, there is no available panel of markers for defining the inflammatory stages of obesity and/or a physiologically helpful cut-off value for inflammation [16].

Definitive work has shown a direct association between the increase in obesity class and the presence of obesity-related comorbidities with the elevated levels of inflammatory biomarkers [17]. The overexpression of proinflammatory cytokines is considered to be the link between obesity-induced inflammation and endothelial dysfunction [15]. In this sense, TNF-α has received considerable attention as one of the key mediators of inflammation involved in obesity and is found in high concentrations in subjects with obesity than in lean [7,18,19]. Also increased AT expression of TNF-α has been reported [20]. Our results came in concordance with the literature and other previous studies regarding TNF-α levels in class II and III obesity groups. As the majority indicated that women with obesity, BMI ≥ 34.5 kg/m^2^, showed significant increase in TNF-α level [21].

It is generally well established that, in obesity the increased mass of AT simultaneously activates the inflammatory process in the white AT (WAT) itself, in liver and in immune cells. Causing increased levels of circulating proinflammatory cytokines, hormone-like molecules and other inflammatory markers, which may show both local and systemic effects [22]. Furthermore, obesity is associated with pathological changes in AT morphology including infiltration of immune cells. Macrophage infiltration of WAT in subjects with obesity increases proportionally to BMI and body fat mass, and this effect is reversible on weight loss [3,23]. The cytokine profile secreted by hypertrophic adipocytes includes TNF-α, IL-6 and MCP-1, all with mainly pro-inflammatory characteristics [24]. However, we noted no statistically significant change in the serum levels of TNF-α in class I obesity. It seems that the concentration of this molecule gradually increases with the obesity level. Peluso and Palmery [25] suggested that inflammation is a consequence rather than a cause of obesity. Additionally, TNF-α is positively correlated with BMI and this correlation could be considered an important factor in obesity pathogenesis [26]. Also, some authors have reported that the expression of TNF-α mRNA in the AT is positively correlated to the body fat, insulin and TG levels [27]. On the other hand, Radziavicius et al. [28] study on subjects with obesity (BMI ≥ 35–40 kg/m^2^) awaiting bariatric surgery showed no significant change at TNF-α level.

IL-6 stands out among the mediators involved in the pathogenesis of obesity. Our results agree with the fact that circulating levels of inflammatory markers are elevated in human subjects with obesity and associate with obesity-related parameters [3,18]. IL-6 is produced by AT, fibroblasts, endothelial cells, macrophages, monocytes and lymphocytes, and contributes to acute phase reactions and chronic inflammatory processes [23]. Obesity enhances gene expression of IL-6 and its receptor in the human subcutaneous AT, which correlates positively with the local expression of several inflammatory markers. Unlike TNF-α, IL-6 principally secreted in an endocrine (systemic) fashion [29] and the expanding AT in obesity may contribute high levels of IL-6 in the circulation [20,28]. Consequently, the accumulation of excessive macronutrients in the ATs in obesity, stimulates the release of inflammatory mediators like TNF-α and IL-6, causing the predisposition of the endothelium toward a proinflammatory state gearing to endothelial dysfunction [15]. Although the levels of IL-6 have been clearly demonstrated to be increased in relation to obesity, we reported that class II obesity group did not have a statistically significant change in levels of that cytokine. According to Nguyen et al. [17], the strongest association between the change in biomarker concentration and obesity was observed among those in obesity class III. Comparable findings in a study of Khaodhiar et al. [29], reported that the serum levels of IL-6 best indicate the intensity of the systemic inflammation that develops with increasing levels of obesity, and IL-6 levels was positively correlated with BMI only in subjects with morbid obese. On the other side, Feitosa et al. [21] indicated that concentrations of IL-6 in plasma did not show significant differences between women with obesity and controls.

Individuals with obesity present high concentrations of CRP, an acute phase inflammatory marker mainly produced in the liver, and this process is regulated predominately by IL-6 [18,28,30]. Our elevated CRP levels among the three obesity classes confirmed the link between inflammation and increased CRP levels in circulation of individuals with obesity. In a large cohort of 674, Paepegaey et al. [31] showed that CRP increased significantly with BMI (studying five different categories of BMI ranging from 35–40 to 47.4 kg/m^2^), suggesting that expanded and inflamed AT is the main source of increased circulating CRP in populations with obesity, through the production of cytokines, which stimulate CRP release from the liver. Likewise, Zimmermann et al. [32] indicated that per every 10% increase in BMI, the CRP level increased by 19–20%. On the other hand, Ramdas and Jella [33] reported that higher BMI is associated with higher CRP concentrations, pointing to that the levels of CRP were within the normal ranges till class I obesity individuals while class II and III subjects exhibit elevated level of CRP.

Very limited studies have precisely determined serum IL-12 levels in subjects with obesity and the present study may be the first regarding the obesity classes among females. IL-12, is a proinflammatory cytokine composed of a 40-kDa (p40) subunit and a 35-kDa (p35) subunit. p40 might have an independent role in initiating the immune response [34]. This heterodimeric class I helical cytokine is mainly produced by dendritic cells (DCs) and macrophages, and influences differentiation of T helper 1 (Th1) immune cells [19]. In the context of immune cells interaction in AT in obesity, Liu and Nikolajczyk [16] indicated that obese AT exhibited a shift in macrophage polarization from a simplistically designated anti-inflammatory M2-like (F4/80−) to a pro-inflammatory M1-like (F4/8+) macrophage phenotype, the latter is considered a main contributor to AT inflammation in obesity. In addition, CD11c^+^ DCs secrete IL-12 and IL-18 which further polarize CD4^+^ T cells to Th1 cells, that interact with adipocytes. Then, adipocytes communicate with the invariant natural killer T (iNKT) cells to produce IL-2 and other cytokines that generally counter AT inflammation.

Plasma levels of IL-12 family members were elevated with obesity, diabetes and metabolic syndrome, while their cellular origin has not been fully determined [35]. IL-12 induces the expression of cytotoxic mediators and increased production of cytokines, especially IFN-γ, in NK cells, T cells, and natural killer T cells. The study of Kim et al. [36], suggested that IL-12 levels in addition to NK cell activity could be better indicators for predicting individuals who are at early immune risk than serum levels of TNF-α, IL-6 and IL-1β. Along with our results, the study of obesity in the Mexican adult population revealed that, overweight and subjects with obesity (21 women with BMI ≥ 30 kg/m^2^) had higher levels of IL-12 than a normal-weight group. Additionally, serum levels of IL-12 correlated with systemic low-grade inflammation, BMI and the grade of abdominal obesity [19]. IL-12 could be involved in the development of obesity-associated co-morbidities, especially atherosclerosis. Nikolajuk et al. [34] data indicate that the IL-12/IL-12p40 system may be associated with lipid abnormalities in subjects with obesity. The altered cytokine secretion in obesity patients depend on the amount of visceral rather than general obesity [37]. In consistent, Schmidt et al. [38] reported that obesity group had significantly elevated serum concentrations of IL-5, IL-10, IL-12, IL-13, IFN-γ and TNF-α compared with the normal group. Notably, the distribution of AT may be a factor in alterations of cytokine levels and may also relate obesity to highly prevalent comorbidities such as insulin resistance (IR), diabetes, atherosclerosis, sleep disturbances and asthma.

Besides its direct impact on inflammation, obesity can also affect the endothelial cell markers. The cell adhesion molecules (CAMs) are membrane receptors glycoproteins that play an important role in the adhesion of cells to extracellular matrices and to endothelial surfaces [39]. Endothelial dysfunction is a multifactorial process in obesity. The increased production of a number of pro-inflammatory cytokines and chemokines triggers local effects on the endothelium. Damage on the injured endothelium leads to an increased production of CAMs and vascular permeability, which ultimately are translated into an increase in monocyte infiltration and accumulation of macrophages, contributing extensively to vascular endothelial dysfunction observed in obesity and its related metabolic syndromes [8,15]. However, the current knowledge still misses the grade of obesity in which endothelial dysfunction begins. Considering obesity as a multiple grade disease, Kraemer-Aguiar et al. [40] have hypothesized that an increasing impairment of endothelial function occurs from lean to severe obese subjects.

Indeed, several studies have established an association between metabolic syndrome (MetS) with inflammatory markers and the loss of permeability, vasoconstriction and vasodilatation [41–43]. The attachment of monocytes and lymphocytes to endothelial cells, is a key factor in atherosclerosis, and appears under the effect of adhesion molecules [44]. The adhesion molecules sICAM-1 and sVCAM-1, as markers of vascular inflammation, were significantly increased in the females with obesity compared with controls and subsequently may contribute to cardiovascular outcomes [41,45,46]. Thus, a large number of new inflammatory biomarkers are currently being studied as possible mediators of inflammation. Our data indicated elevated levels of sVCAM-1 among the three obesity groups. Similarly, Glowinska et al. [42] emphasized that biomarkers of endothelial dysfunction (sICAM-1 and sVCAM-1) are elevated in obesity individuals. In this regard, the inflammatory mediators are activated by nuclear factor κB (NF-κB) signaling and regulates the expansion of endothelial cells via increasing the expression of ICAM-1, resulting in the relocation of leukocytes and inducing the progression of atherosclerosis [47]. Also, ICAM-1, VCAM-1 and E-selectin intervene with inflammatory cytokines of the vascular wall to promote extravasations and subsequent endothelial dysfunction [15]. VCAM-1 has a unique pattern of regulation. It is not expressed in basal conditions but is rapidly induced by pro-atherosclerotic conditions. It is induced by IL-4 and high concentrations of ROS oxidized LDL-C, while the expression of ICAM-1 is regulated by TNF-α [8]. Our results seem to be consistent with this hypothesis regarding the elevated ICAM-1 levels in class III obesity group, while no statistically significant change in initial stages of obesity (obesity class I and II).

## Conclusion

The associations between weight and vascular health, besides their influential role in the development of obesity related diseases, are already well-documented in the literature. However, there was a need to understand the variability of inflammation and endothelial dysfunction markers among the obesity classes, as the obesity rate is still mounting, to pinpoint more description of the inflammatory network in each class. Herein, data presented in our study highlight the alterations in the serum levels of TNF-α, CRP, IL-6, IL-12, sVCAM-1 and sICAM-1 with the progression of obesity among Egyptian females. First, our data strongly support the hypothesis that, with the progression of obesity, a subclinical inflammatory reaction has a role in the pathogenesis of obesity and emphasize the elevation of endothelial dysfunction in individuals with obesity. Second, to our knowledge, this report is the first study to demonstrate IL-12 among females with obesity classes I–III. We bring attention that, class I and class III exhibited elevated levels of IL-12. Third, the outcome of such differences in inflammatory and endothelial dysfunction biomarkers among obesity classes could personalize and improve the obesity interventions, in addition to creating a window for new therapeutic strategies to obesity and/or its associated metabolic diseases. Finally, future studies with larger sample size should be done to validate the reliability of our results.

## Abbreviations

AT: adipose tissue
BMI: body mass index
CAM: cell adhesion molecule
CRP: C-reactive protein
CVD: cardiovascular disease
DC: dendritic cell
ELISA: enzyme-linked immunosorbent assay
IFN-γ: interferon gamma
IL: interleukin
IR: insulin resistance
LDL-C: low-density lipoprotein - cholesterol
MCP-1: monocyte chemoattractant protein-1
ROS: reactive oxygen species
sICAM-1: soluble intercellular adhesion molecule 1
sVCAM-1: soluble vascular adhesion molecule 1
Th1: T helper 1
TNF-α: tumor necrosis factor-α
WAT: white AT
